# Reversible dementias

**Published:** 2009-01

**Authors:** Manjari Tripathi, Deepti Vibha

**Affiliations:** Department of Neurology, All India Institute of Medical Sciences, New Delhi, India

**Keywords:** Dementia, reversible, treatable

## Abstract

In recent years, more attention has been given to the early diagnostic evaluation of patients with dementia which is essential to identify patients with cognitive symptoms who may have treatable conditions. Guidelines suggest that all patients presenting with dementia or cognitive symptoms should be evaluated with a range of laboratory tests, and with structural brain imaging with computed tomography (CT) or magnetic resonance imaging (MRI). While many of the disorders reported as ‘reversible dementias’ are conditions that may well be associated with cognitive or behavioral symptoms, these symptoms are not always sufficiently severe to fulfill the clinical criteria for dementia. Thus, while the etiology of a condition may be treatable it should not be assumed that the associated dementia is fully reversible. Potentially reversible dementias should be identified and treatment considered, even if the symptoms are not sufficiently severe to meet the clinical criteria for dementia, and even if partial or full reversal of the cognitive symptoms cannot be guaranteed. In the literature, the most frequently observed potentially reversible conditions identified in patients with cognitive impairment or dementia are depression, adverse effects of drugs, drug or alcohol abuse, space-occupying lesions, normal pressure hydrocephalus, and metabolic conditions land endocrinal conditions like hypothyroidism and nutritional conditions like vitamin B-12 deficiency. Depression is by far the most common of the potentially reversible conditions. The review, hence addresses the common causes of reversible dementia and the studies published so far.

## INTRODUCTION

As clinicians have approached dementia with therapeutic nihilism, there has been limited interest in extensive differential diagnosis beyond the exclusion of the traditional reversible dementias. Nevertheless, all dementias are treatable through nursing, social work, and palliative medical interventions. Early recognition of a progressive degenerative dementia gives patients the opportunity to express or clarify end-of-life plans while judgment and personality are largely intact, and enables the family to plan for the financial aspects of caring for the affected person.[1] It is estimated that 24.3 million people worldwide have dementia, with 4.6 million new cases of dementia every year (one new case every 7 s). The number of people affected will double every 20 years to reach 81.1 million by 2040.[2] Dementia is a clinical diagnosis whose evaluation involves assessment of the presenting problem; history about the patient that is provided by an informant (someone who knows the patient, usually a family member); complete physical and neurologic examination; evaluation of cognitive, behavioral, and functional status; and laboratory and imaging studies.

## REVERSIBLE DEMENTIA: IS IT DEMENTIA?

For a clinical diagnosis of dementia, deficits in memory and in at least one other cognitive domain must be documented, the deficits should interfere with occupational or social functioning, and there must be evidence of a systemic or brain disorder that may be the primary cause of the cognitive deficits (American Psychiatric Association, 1993). The American Academy of Neurology practice parameter recommends structural neuroimaging, which may include CT or MRI, and screening for depression, vitamin B12 deficiency, and hypothyroidism. Based on these criteria the screening for syphilis without risk factors is not justified. Neuroimaging screening evaluations are recommended to detect conditions such as subdural hematomas, cerebral infarcts, cerebral tumors, and normal pressure hydrocephalus [Table 1].

**Table 1:** DSM IV criteria for dementia[3]

Development of multiple cognitive deficits manifested by both: 1. Memory impairment (impaired ability to learn new information or to recall previously learned information) 2. One (or more) of the following cognitive disturbances: - aphasia (language disturbances) - apraxia (impaired ability to carry out motor activities despite intact motor function) - agnosia (failure to recognize or identify objects despite intact sensory function) - disturbance in executive functioning (i.e., planning, organizing, sequencing, abstracting) Cognitive deficits in criteria A1 and A2 each cause significant impairment in social or occupational functioning and represent a significant decline from a previous level of functioning Deficits do not occur solely during a delirium Deficits not due to psychiatric disease (major depression, schizophrenia)

## HOW COMMON?

The reported frequency of dementia due to potentially reversible causes varies from 0 to 23%.[4–6] Commonest among these causes are alcohol and medication related dementia, depression induced cognitive impairment, surgical brain lesions such as normal pressure hydrocephalus [NPH], tumors and chronic subdural hematomas, metabolic disorders such as hypothyroidism, hypoparathyroidism, vitamin B12 deficiency and central nervous system (CNS) infections such as neurosyphilis and HIV [Table 2].[7]

**Table 2:** Prevalence of potentially reversible conditions in patients with cognitive impairment or dementia

Type of study	Reference	Setting	Number of patients	Potentially-reversible condition*	Partly reversed*	Fully reversed*	Not reversed*
Systematic review (33 studies)	Clarfield 1988	Various	2889	15.2	NA	NA	NA
Systematic review (16 studies)	Weyting *et al*. 1995	Various	1551	13.2	9.3	1.5	2.4
Prospective study	Hejl *et al*. 2001	Outpatient neurology-based memory clinic	785	20	NA	NA	NA
Meta analysis	Clarfield 2003	Various	5620	9	0.29	0.31	8.4

* Figures are in percentages

The Indian study by Srikanth found reversible causes, especially neuroinfections and vitamin B12 deficiency to account for 18% of all dementias.[8]

## WHAT ARE THE REVERSIBLE CAUSES?

There are several clearly reversible causes of dementia that are remembered by the mnemonic DEMENTIA:

Drugs (any drug with anticholinergic activity), emotional- depression, metabolic (hypothyroid), eyes and ears declining, normal pressure hydrocephalus tumor or other space-occupying lesion, infection (syphilis, AIDS), anemia (vitamin B12 or folate deficiency).

The major syndromes with progressive dementia include Alzheimer disease (AD), vascular dementia (VaD), dementia with lewy bodies (DLB) and frontotemporal dementia (FTD) [Table 3].

**Table 3:** Causes of potentially reversible cognitive impairment or dementia

Neurosurgical conditions	Neuroinfections and inflammations	Metabolic conditions	Others
Subdural hematoma	Meningitis (tubercular, fungal, malignant)	Hypo and hyper thyroidism,	Depression
		Hashimotos encephalits	
Normal pressure hydrocephalous	Encephalitis (limbic, HIV, herpes)	Hypo and hyper parathyroididsm	Epilepsy
Intracranial tumors	Cerebral vasculitis	Pituitary insufficiency	Drugs and toxins
Intracranial empyema or abscess	Neurosyphilis	Hypercalcemia	Alcohol abuse
	Lyme’s disease	Cushing’s disease	Sleep apnea
	Whipple’s disease	Addison’s disease	Limbic encephalitis (neoplastic/ autoimmune)
	Sarcoidosis	Hypoglycemia	
		Vitamin deficiencies (B1, B6, B12, and folate)	
		Chronic liver failure	
		Chronic respiratory failure	
		Chronic renal failure	
		Wilson’s disease	

### Neuroinfections and inflammations

AIDS-dementia complex, HIV encephalopathy, or HIV-associated dementia is a neurologic complication of acquired immunodeficiency syndrome, eventually occurring in one fourth of patients who have AIDS. It typically occurs in the later stages of HIV infection and has diminished since the introduction of highly active antiretroviral therapy.[9] Cryptococcus and JC virus infections typically present with meningitis or progressive focal neurologic deficits, respectively; however, they also can present with rapid progression of dementia.

Spirochete infections are unusual causes of cognitive impairment but important to consider as they are treatable. No workup for dementia, including rapidly progressive dementia (RPD), is complete without an evaluation for CNS infection with Treponema pallidum, or neurosyphilis. Cognitive dysfunction is the most common neurologic syndrome, although usually a late complication, of syphilis. Lyme disease is a systemic infection with the spirochette, Borrelia burgdorferi, which is transmitted to people from a tick bite. Neurologic manifestations are rare in Lyme disease but can include cranial nerve palsy, meningitis, polyradiculopathy, depression, psychosis, and dementia.

Whipple’s disease is a rare bacterial (Tropheryma whippelii) infection, involving many organ systems that can present as a neuropsychiatric syndrome that, although typically insidious, can progress rapidly over months. More than 80% of the cases have been diagnosed in men. Although the age range varies from childhood to the elderly, onset typically is in the fifth through seventh decades, with an approximate mean age of onset of 50. Clinical presentation is varied. It most commonly presents as a malabsorption syndrome with diarrhea, abdominal pain, weight loss, arthralgias, wasting, fever, and lymphadenopathy; but as many as 15% of cases do not exhibit gastrointestinal symptoms. CNS involvement occurs in 5 to 45% of cases, with 5% of cases having neurologic presenting symptoms.[10]

### Metabolic and toxic causes

Several endocrinal disorders and vitamin deficiencies can masquerade as dementia and need to be investigated, especially in young and rapidly progressive dementias. Several toxins can cause RPD. Exposure to heavy metals, such as arsenic, mercury, aluminum, lithium, or lead, can lead to cognitive decline, particularly after acute exposure. Most cases of acute exposures result in florid encephalopathies that progress over hours to days and thus would not be confused with rapidly progressive dementias, which progress over weeks to months. Manganese toxicity, found usually in miners, can present with significant Parkinsonism.[11] Bismuth is a metal used to treat gastrointestinal disorders, principally peptic ulcer disease and diarrhea. Bismuth intoxication, typically caused by overdosing on bismuth-containing products, such as Pepto-Bismol, can cause a disorder mimicking Creutzfeldt-Jakob disease (CJD). Patients initially manifest with apathy, mild ataxia, and headaches, which progress to myoclonus, dysarthria, severe confusion, hallucinations (auditory and visual), seizures, and, in severe cases, even death.[12] Blood levels of bismuth, greater than 50 mg/L, are considered in the toxic range. The condition usually is reversible; however, extremely prolonged use can result in permanent tremors.

### Drugs and unusual causes

Steroid-induced cognitive effects have long been conflated with the more affective behavioral manifestations of the steroid psychosis syndrome. A 1979 review of 13 patients with steroid psychosis found marked distractibility in 79%, intermittent memory impairment in 71%, and persistent memory impairment in 7% of patients.[13] Varney *et al*. first coined the term “steroid dementia” when they described the effects of long-term glucocorticoid (GC) use in 1,500 patients.[14] However, steroid induced cognitive decline is overlooked as a reversible factor.[15,16]

Other uncommon causes quoted as reversible causes of dementia have been spontaneous intracranial hypotension, idiopathic hypereosinophilic syndrome.[17,18]

### Nonorganic (psychiatric) causes of rapidly progressive dementia

Pseudodementia, resulting from depression, occurs in patients who have a past history of major depression. There usually are signs that patients are severely depressed, and cognitive dysfunction, particularly on testing, is found to be the result of decreased effort. Many of the features of patients who have true dementia are seen in atypical psychiatric disorders, including personality disorders, conversion disorders, psychosis and malingerers, and a full assessment is required to rule out potentially treatable or organic disorders. These cases can have many of the features of a true dementia. Furthermore, psychiatric features may be an early symptom of many neurodegenerative conditions, including CJD, diffuse lewy body disease (DLB), corticobasal ganglionic degeneration (CBD), and others.[19]

### Vascular etiologies

Thrombotic thrombocytopenic purpura can cause microangiopathic thromboses producing global cerebral ischemia, resulting in an encephalopathy. Hyperviscosity syndromes from blood dyscrasias, such as polycythemia, or gammopathies, such as Waldenstrom’s macroglobulinemia, can present as dementia by causing global cerebral microvessel ischemia.[20]

CNS vasculitis can also present as dementia. A vasculitis may be limited to the CNS without any systemic or peripheral nervous system signs or may present initially as a systemic disorder with accompanying fever, weight loss, rash, neuropathy, and other organ involvement. Urinalysis may contain red cells as a sign of renal involvement. Ophthalmologic examination may identify uveitis, scleritis, or signs of ophthalmic artery vasculitis. If a rash is present, a skin biopsy can be diagnostic. There may be signs of a hemolytic anemia. A basic rheumatologic screen may include erythrocyte sedimentation rate (ESR), C-reactive protein (CRP), complement (C3), complement (C4), total complement (CH50), antinuclear antibody (ANA), rheumatoid factor (RF), anti-SSA, anti-SSB, perinuclear antineutrophil cytoplasmic autoantibodies (p-ANCA), and cytoplasmic ANCA (c-ANCA) with other testing depending on results of this initial screen. Serologic testing likely is abnormal in systemic forms of vasculitis, but in primary CNS vasculitis, patients typically have normal nonspecific tests, such as ESR, ANA, and CRP.[21]

## HOW TO INVESTIGATE?

In a patient with dementia, a detailed history can save us from a number of redundant investigations. However, to rule out potentially reversible causes, especially if there is a high index of suspicion from the presentation and clinical signs elicited on examination, further elaborate investigations may be ordered.

An algorithm that can be followed while investigating for reversible causes is by etiology, therefore

### 

#### To rule out infections -

- Viral PCRs and cultures
- Bacterial, fungal, AFB stains and cultures;
- Whipple’s PCR

#### To rule out autoimmune-

- ESR, CRP, C3, C4, CH50, ANA, RF, anti-SSA, anti-SSB, Anti-dsDNA, anti-Smith, P-ANCA, C-ANCA, antiendomysial and anti-gliadin IgA and IgG, ACE, paraneoplastic and other auto-antibodies (e.g., Anti-GAD 65, VGKC, neuropil, etc…)

#### To rule out malignancy-

- CT scan body with and without contrast
- Whole body PET scan
- CSF Cytology and Flow cytometry
- Serum LDH, tumor markers (PSA, CEA, etc.)
- Mammogram

#### To rule out vascular causes-

- Hypercoagulability testing; coagulation profile Echocardiogram; carotid ultrasound
- Cerebral angiogram, meningeal biopsy

#### To rule out toxic and metabolic causes -

- 24 h urine heavy metal for lead, arsenic, and mercury, bismuth, aluminum, lithium
- Serum Vitamins B12 and E, homocysteine, methylmalonic acid
- Serum copper and ceruloplasmin; 24h urine copper
- Exposure history

## CONCLUSIONS

It is mandatory that all potentially-reversible conditions are investigated for in any patient presenting with cognitive complaint, as this is frequently encountered in patients with cognitive impairment or dementia; in some they are the cause of the dementia, in others they are a comorbidity to a progressive dementing disorder.[22] Numerous potentially reversible conditions may be associated with or even present with cognitive impairment. Some are rare, yet treatable (e.g., Wilson’s disease).

Other conditions are more common, but may only occasionally present with dementia in the absence of other neurological symptoms (e.g., space-occupying lesions). Finally, some are very common and should be looked for in all patients with dementia (e.g., depression, vitamin deficiency). Most reversible conditions are easily identified by a careful history, physical examination, psychiatric evaluation, brain CT or MRI, and routine laboratory tests.
